# Functional MRI assessment of the lungs in fetuses that deliver very Preterm: An MRI pilot study

**DOI:** 10.1016/j.ejogrb.2023.12.015

**Published:** 2024-02

**Authors:** Carla L. Avena-Zampieri, Jana Hutter, Alena Uus, Maria Deprez, Kelly Payette, Megan Hall, Mona Bafadhel, Richard E.K. Russell, Anna Milan, Mary Rutherford, Andrew Shennan, Anne Greenough, Lisa Story

**Affiliations:** a Department of Women and Children’s Health King’s College London, United Kingdom; b Centre for the Developing Brain, School of Biomedical Engineering & Imaging Sciences, King’s College London, United Kingdom; c Fetal Medicine Unit, Guy’s and St Thomas’ NHS Foundation Trust, United Kingdom; d Neonatal Unit, Guy’s and St Thomas’ NHS Foundation Trust, United Kingdom; e Department of Biomedical Engineering, School of Biomedical Engineering & Imaging Sciences, King’s College London, United Kingdom; f King’s Centre for Lung Health, School of Immunology and Microbial Sciences, Faculty of Life Sciences and Medicine, King’s College London, London, United Kingdom

**Keywords:** Lung development, Fetal MRI T2*, Prenatal prediction

## Abstract

**Objectives:**

To compare mean pulmonary T2* values and pulmonary volumes in fetuses that subsequently spontaneously delivered before 32 weeks with a control cohort with comparable gestational ages and to assess the value of mean pulmonary T2* as a predictor of preterm birth < 32 weeks’ gestation.

**Methods:**

MRI datasets scanned at similar gestational ages were selected from fetuses who spontaneously delivered < 32 weeks of gestation and a control group who subsequently delivered at term with no complications. All women underwent a fetal MRI on a 3 T MRI imaging system. Sequences included T2-weighted single shot fast spin echo and T2* sequences, using gradient echo single shot echo planar sequencing of the fetal thorax. Motion correction was performed using slice-to-volume reconstruction and T2* maps generated using in-house pipelines. Lungs were manually segmented and volumes and mean T2* values calculated for both lungs combined and left and right lung separately. Linear regression was used to compare values between the preterm and control cohorts accounting for the effects of gestation. Receiver operating curves were generated for mean T2* values and pulmonary volume as predictors of preterm birth < 32 weeks’ gestation.

**Results:**

Datasets from twenty-eight preterm and 74 control fetuses were suitable for analysis. MRI images were taken at similar fetal gestational ages (preterm cohort (mean ± SD) 24.9 ± 3.3 and control cohort (mean ± SD) 26.5 ± 3.0). Mean gestational age at delivery was 26.4 ± 3.3 for the preterm group and 39.9 ± 1.3 for the control group. Mean pulmonary T2* values remained constant with increasing gestational age while pulmonary volumes increased. Both T2* and pulmonary volumes were lower in the preterm group than in the control group for all parameters (both combined, left, and right lung (p < 0.001 in all cases). Adjusted for gestational age, pulmonary volumes and mean T2* values were good predictors of premature delivery in fetuses < 32 weeks (area under the curve of 0.828 and 0.754 respectively).

**Conclusion:**

These findings indicate that mean pulmonary T2* values and volumes were lower in fetuses that subsequently delivered very preterm. This may suggest potentially altered oxygenation and indicate that pulmonary morbidity associated with prematurity has an antenatal antecedent. Future work should explore these results correlating antenatal findings with long term pulmonary outcomes.

## Highlights

• Application of deformable slice-to-volume reconstruction is feasible for MRI characterisation of preterm lung development.
• Mean T2* values were lower in the preterm cohort than in controls.
• Alterations in pulmonary development associated with preterm birth may commence in the antenatal period.

## Background

Preterm birth (PTB) is a common obstetric complication, affecting 8 % of all deliveries in the UK [1] and is associated with significant morbidity and mortality [2]. The severity of adverse outcomes for the infant is inversely correlated with the gestational age (GA) at delivery, with the most significant morbidity occurring where delivery is very preterm, before 32 weeks gestation [3]. Although a number of organ systems can be affected by PTB (5 % of individuals develop cerebral palsy [4], 5–12 % necrotizing enterocolitis [5] and 12.6 % retinopathy of prematurity [6]) respiratory complications are considerable, affecting up to 15.8 % of very preterm infants [7], [8].

The most common acute term respiratory complication associated with spontaneous preterm birth (sPTB) is respiratory distress syndrome (RDS), attributable to a lack of pulmonary surfactant [9]. The incidence of RDS is inversely related to gestational age as 98 % of babies born at 24 weeks present with RDS while at 34 weeks, the incidence falls to 5 %. One study evaluated the rate of RDS among live births < 32 weeks’ gestation and found a rate of 113.2 per 1000 live births [10]. While the management of RDS has drastically improved after the introduction of intratracheal surfactant replacement, the incidence of long-term respiratory complications like bronchopulmonary dysplasia (BPD) has not markedly changed. BPD is a chronic condition in which alveolar and microvascular development of the peripheral lung is disrupted, requiring prolonged respiratory support and supplementary oxygen. This condition is more prevalent in very prematurely born infants [11], [12], with a reported incidence ranging from 4 % to 15.8 % of infants born between 24 and 32 weeks’ gestation across various countries [7]. BPD additionally predisposes to longer term pulmonary sequelae such as asthma [13] and an increased risk of hospitalisation, particularly due to respiratory viral infections [14]. Compromised pulmonary function has also been reported in preterm born adolescents and adults and is associated with reduced exercise tolerance.

While mechanisms contributing to BPD are not completely understood, multiple antenatal and postnatal factors are known to contribute to its development. There is evidence that infection and inflammation may play a role in the aetiology of PTB [15] and infection and inflammatory cytokines have been also shown to alter pulmonary development [16]. It is therefore plausible that alterations in lung development associated with prematurity may commence prior to preterm delivery. Indeed, MRI studies have indicated that fetal lung volumes are reduced in fetuses who then delivered prematurely [17], [18].

The use of advanced fetal MRI techniques might provide a more in depth understanding of the antenatal factors associated with lung development in preterm babies. T2* relaxometry for example, is based on the different paramagnetic properties expressed by oxygenated and deoxygenated haemoglobin, and can be used to facilitate assessment of tissue function in addition to structure. This approach can therefore provide an indirect assessment of oxygenation/perfusion of a selected region of tissue. Application of these techniques for evaluation of fetal lung development in pregnancies at high-risk of sPTB may give insight into antenatal antecedents of respiratory morbidity associated with prematurity.

This study aims to 1) assess mean pulmonary T2* values and pulmonary volumes in fetuses that subsequently spontaneously deliver before 32 weeks of gestation and compare findings with a group of control fetuses that subsequently deliver at term 2) assess the value of mean pulmonary T2* as a predictor of preterm delivery before 32 weeks of gestation.

## Methods

A retrospective study was performed comparing pregnancies that subsequently delivered spontaneously prior to 32 weeks of gestation from uncomplicated pregnancies that delivered at term. Datasets were selected from three ethically approved studies: (REC: 16/LO/1573, IRAS 201609), (19/LO/0736, IRAS 253500), (REC: 21/SS/0082, IRAS 293516). All studies were undertaken at St Thomas’ Hospital over a five-year period from 2018 to 2023.

Preterm datasets were selected where women had delivered spontaneously < 32 weeks of gestation. The women had been recruited to the above three studies because they were at high risk of preterm delivery. Selection criteria included: singleton pregnancy, high risk of sPTB (more than > 50 % risk of delivery < 32 weeks of gestation calculated using a validated algorithm encompassing maternal history, quantitative cervico-vaginal fibronectin levels and cervical length obtained by transcervical ultrasound [19]) or preterm premature rupture of the membrane (PPROM) prior to 32 weeks who were not in active labour. The control group was identified from the same studies, where the pregnancy was low risk throughout and delivery occurred after 37 weeks of gestation. Exclusion criteria for both groups included: maternal medical conditions, pregnancy complications such as gestational diabetes or pre-eclampsia, fetal congenital or genetic abnormalities, inability to give informed consent, contraindications to MRI such as claustrophobia or a recently sited metallic implant or incomplete outcome data. Cases with a birthweight centile < 3rd or > 97th centile (calculated using the INTERGROWTH centiles) [20] were also excluded given they may have had additional underlying aetiologies.

Imaging was performed on a clinical 3 T Philips MRI scanner (Phillips Best) using a 32-channel cardiac coil and with women in a semi-supine position [21].

Anatomical imaging of the fetal thorax was obtained using a T2-weighted single-shot turbo spin echo sequence acquired in three orthogonal planes focused on the fetal body. An additional sequence, focused on the whole uterus in the coronal plane was also obtained [22]. A main magnetic field (B0) map was acquired to enable image based (2nd order) shimming [23] using an in-house pipeline. T2* images were yielded using a multi-echo gradient echo single-shot echo planar sequence with the following parameters: 3 mm^3^ resolution, five echo times [13.8 ms / 70.4 ms / 127.0 ms / 183.6 ms / 240.2 ms], repetition time (TR) = 3 s, parallel imaging SENSE factor = 3, flip angle = 90°. The field of view was set to 360 mm × (320–400) mm × (60–120) mm using a coronal imaging plane aligned to the scanner co-ordinates, in order to encompass the whole thorax and minimise artefacts from parallel imaging reconstruction techniques. The imaging protocol was completed within one hour in all cases and women were offered a break halfway through the examination.

An in-house Python script was used to generate T2* maps using mono-exponential decay fitting [22]. Deformable slice to volume reconstruction (DSVR) [24], another in-house developed pipeline, was used to correct both in- and out-of-plane local deformations of fetal organs caused by bending/stretching motion. This technique has previously been validated in non-rigid motion correction of fetal MRI based on a hierarchical DSVR scheme to allow high resolution reconstruction of the fetal body and placenta [25]. Stacks with satisfactory quality with complete data for both lungs were selected.

**Fig. 1:**
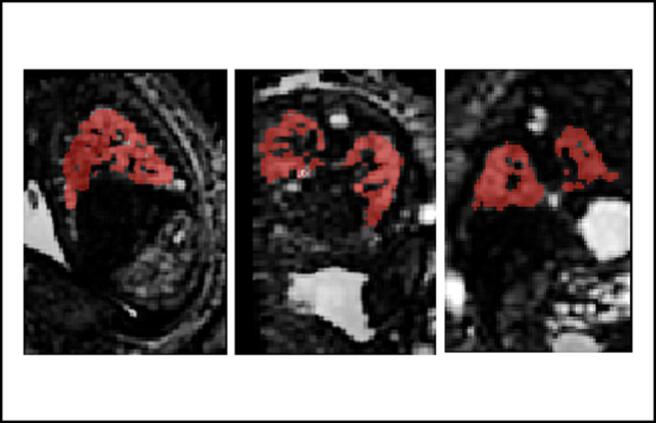
Manual lung segmentation of control dataset at 25 + 4 weeks of pregnancy in the sagittal, axial and coronal on T2* maps after applying deformable slice to volume reconstruction.

Pulmonary tissue segmentation was performed using the computational software 3D-slicer [26] on 3D reconstructed motion-corrected images. Manual pulmonary segmentation was performed by an experienced operator (CAZ) carefully avoiding any non-pulmonary tissue such as vasculature, maternal structures, and amniotic fluid (Fig. 1). Good intra and inter observer correlation had previously been confirmed [27].

Mean T2* values, lung volumes and lacunarity scores (reflective of changes in granularity and heterogeneity of tissue [28]) were generated using a purpose-built Python script [22], [29]. Cases were excluded where data were found to be corrupted or with excessive motion.

Delivery details and maternal and neonatal outcome data were collected from medical records until discharge from hospital. Neonatal outcome parameters assessed were: GA at delivery, birthweight, birthweight centiles, sex of infant, need for neonatal unit admission, numbers of days of invasive ventilation, need for continuous positive airway pressure, supplemental oxygen, RDS, BPD, pneumothorax, persistent pulmonary hypertension of the newborn (PPHN), sepsis, necrotising enterocolitis (NEC) and intra-ventricular haemorrhage (IVH). Placental histology results were also collected where available to assess for the presence of chorioamnionitis. Placental histology was performed by a specialist perinatal pathologist in accordance with the Amsterdam criteria [30].

### Statistical analysis

All data were first checked for normality using standard distributional plots. Non-normal data were transformed by logging to base 10 and re-checked for normality. Demographic and outcome parameters were compared between groups using the Student’s *t* test where data were continuous and the Chi-squared where categorical. Comparison of pulmonary mean T2* values and lung volumes between the term born and preterm cohorts was therefore undertaken using linear regression, accounting for the effects of gestation, ethnicity, and BMI. Receiver operator curves were generated as a predictor of very PTB < 32 weeks. All statistical analysis was undertaken using SPSS version 28.0.1 (SPSS IBM).

## Results

Thirty-one datasets from the preterm cohort and 79 from the control cohort initially met the criteria for inclusion in the study. Three cases from the preterm and five from the control cohorts were subsequently excluded due to significant motion corruption which could not be corrected by post processing pipelines. Twenty-eight preterm and 74 control datasets were therefore suitable for the final analysis. Details of clinical characteristics of the preterm and term cohorts are summarised in Table 1.

**Table 1:** Clinical characteristics of the preterm and term cohorts.

*Characteristics*	*Preterm cohort (n = 28)*	*Term cohort (n = 74)*	*P*
Maternal age (years)			p = 0.79
Mean (SD)	34 (6.2)	33.9 (4.0)	
Range	22–48	25–45	
BMI (kg/m2)			p = 0.01
Mean (SD)	24.4 (3.4)	22.6 (3.0)	
Range	18.3–30.8	18.0–32.5	
Ethnicity (%)			P < 0.001
White	50 %	88 %	
Mixed	8 %	1 %	
Asian	21 %	8 %	
Black	21 %	3 %	
Parity (%)			p = 0.20
0	57 %	72 %	
1	32 %	25 %	
2	7 %	3 %	
3	4 %	0 %	
GA at MRI (weeks)			p = 0.03
Mean (SD)	24.9 (3.3)	26.5 (3.0)	
Range	19.3–31.1	19.6–31.9	
GA at delivery (weeks)			
Mean (SD)	26.4 (3.3)	39.9 (1.3)	
Range	20.14–32	37.0–42.1	
Birthweight (grams)			
Mean (SD)	989 (4 1 6)	3407(4 1 7)	
Range	375–2020	2485–4400	
Birthweight centiles of live neonates (%)			
0–3	0 %	0 %	
3–10	9 %	3 %	
10–25	13 %	16 %	
25–50	4 %	20 %	
50–75	48 %	26 %	
75–90	26 %	24 %	
90–97	0 %	11 %	
97–100	0 %	0 %	
Sex of baby (%)			p = 0.62
Female	56 %	50 %	
Male	44 %	50 %	
Outcome (%)			
Live birth (survived to discharge)	63 %	100 %	
Intrapartum death	22 %	0 %	
Neonatal death	15 %	0 %	

Of the preterm cohort, 19 had ruptured membranes at the time of imaging, nine had intact membranes of which five had bulging membranes. Of the 19 women with PPROM (confirmed clinically on speculum examination) only two had a normal volume liquor on subsequent ultrasound scans. Twenty-two women received antenatal steroids, 19 prior to or on the day of the MRI scan. The mean time from first corticosteroid administration to MRI was 6 ± 8.2 days (range 0–37) and from corticosteroid administration to delivery was 13.8 ± 11.8 days (range 2–47). Twenty-one of the 22 women with corticosteroids administration received a second dose within 24 h of the first. Three had a third dose at least a week after receiving the initial course.

Placental histology was available in 23 of the 28 preterm cohort cases, with 21 showing evidence of chorioamnionitis, nine of which had additional of funisitis. Thirteen of the 21 participants with a histopathological diagnosis of chorioamnionitis had ruptured their membranes.

There was no relationship between GA and mean pulmonary T2* values in the control group for both lungs combined, left lung and right lung (p = 0.14, p = 0.15, p = 0.14 respectively).

**Fig. 2:**
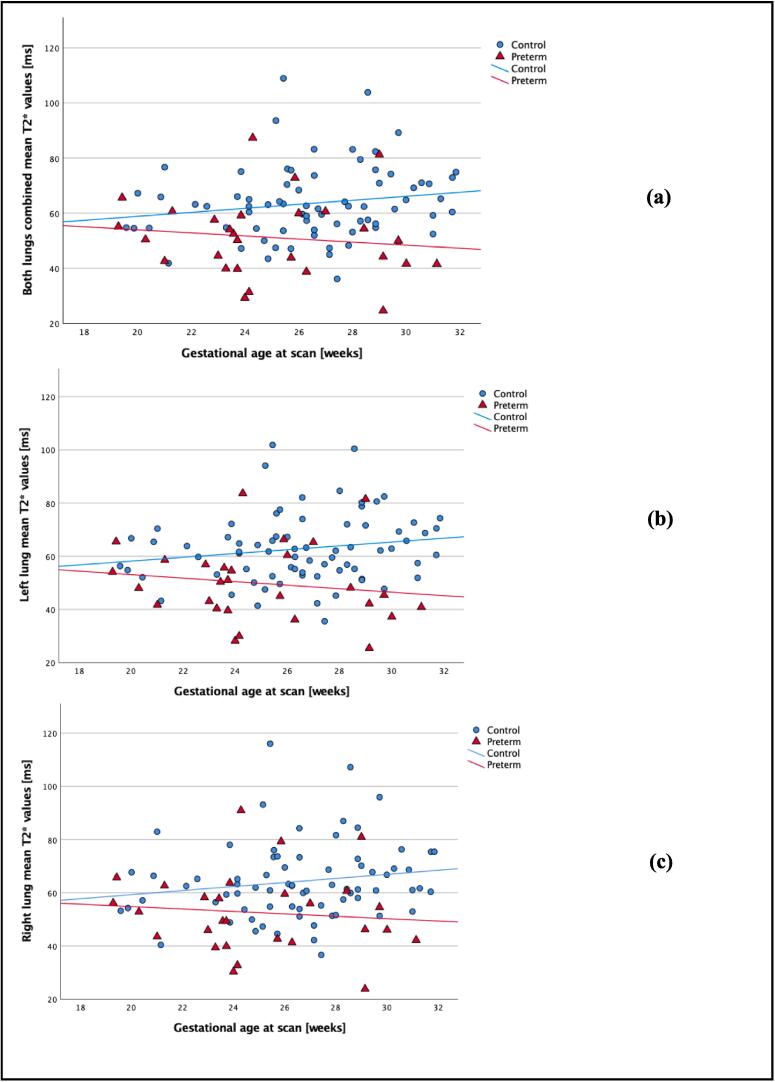
Mean pulmonary T2* values against gestational age at scan in controls (blue dots) and preterm (red triangles). a) represents T2* values in both lungs combined, b) T2* values in left lung and c) T2* values in right lung. Associated subgroups best fit lines are displayed with blue representing the control group and red the preterm. (For interpretation of the references to colour in this figure legend, the reader is referred to the web version of this article.)

Mean pulmonary T2* values were significantly lower in the preterm group than the control group for both lungs combined, the left and right lung (p < 0.001 in all cases see Fig. 2).

**Fig. 3:**
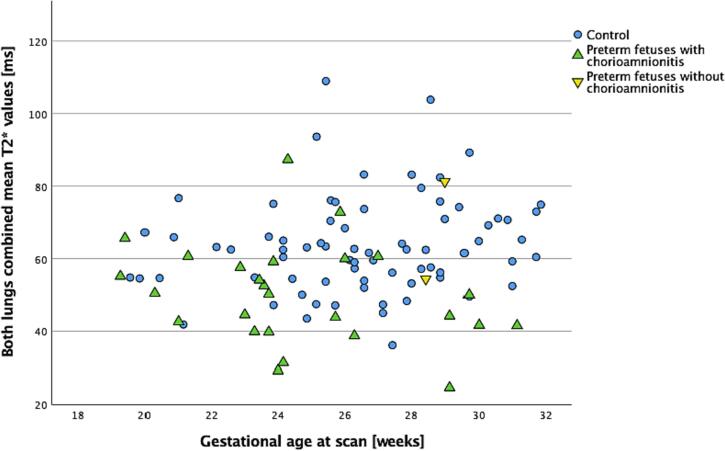
Mean pulmonary T2* values in both lungs in controls (blue dots) vs in both lungs combined in preterm fetuses with chorioamnionitis (green upwards triangles) and without chorioamnionitis (yellow downwards triangles). (For interpretation of the references to colour in this figure legend, the reader is referred to the web version of this article.)

Numbers were too small for statistical analysis but fetuses with and without chorioamnionitis can be seen in Fig. 3.

Numbers were too small in subgroups of women who had intact and ruptured membranes at the time of imaging to assess if this had any impact on mean T2* values.

**Fig. 4:**
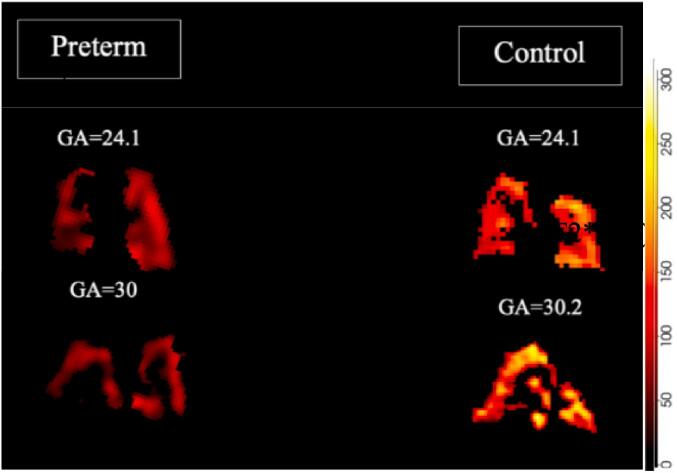
T2* maps in coronal views of fetal lungs in preterm (left) and control (right) pregnancies with a gestational age at scan of 24.1 and 30 weeks.

Examples of T2* maps generated for two preterm cases and gestation matched controls can be seen in Fig. 4. The scale of T2* values is displayed where red colours are low and yellow are high.

**Fig. 5:**
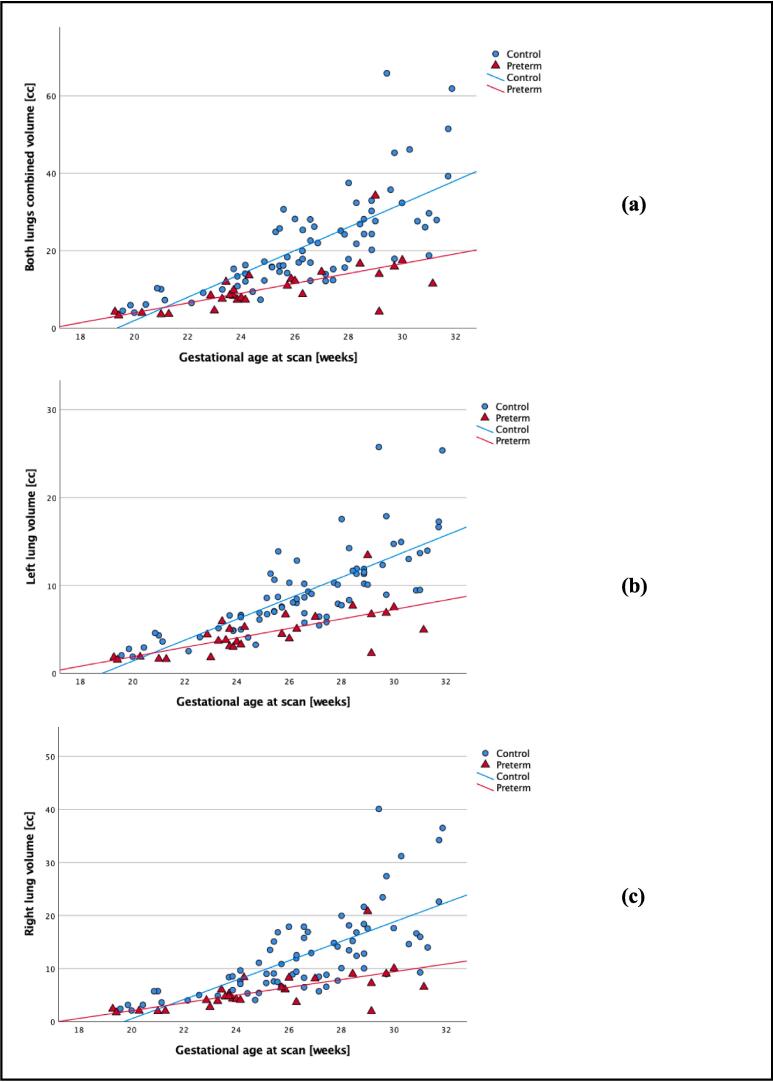
Lung volumes against gestational age at scan in controls (blue dots) and preterm (red triangles). (a) represents lung volumes in both lungs combined, (b) left lung, and (c) right lung. Associated subgroups best fit lines are displayed with blue representing the control group and red the preterm. (For interpretation of the references to colour in this figure legend, the reader is referred to the web version of this article.)

Pulmonary volumes were smaller in the preterm group compared with the control group in both lungs (p < 0.001), left lung (p < 0.001) and right lung (p < 0.001) as seen in Fig. 5.

All neonates from the control group had an uncomplicated postnatal course. In the preterm group 19 developed RDS and six of these were later diagnosed with BPD. Short term neonatal outcomes can be seen in Table 2. Six neonates died intrapartum and four died in the neonatal period. These results are presented to indicate the outcomes of this population appreciating that the numbers are too small for meaningful analysis.

**Table 2:** Outcomes for liveborn infants. Top: Median days and number of cases; CPAP: Continuous Positive Airway Pressure. Bottom: Outcomes for liveborn infants. RDS: respiratory distress syndrome, BPD: bronchopulmonary dysplasia, PPHN: persistent pulmonary hypertension of the newborn, NEC: necrotising enterocolitis, IVH: Intra-ventricular haemorrhage.

	Unit days and respiratory outcomes for liveborn infants (n = 22)						
	Admission to neonatal unit				Non-invasive ventilation		
	Intensive care	High dependency care	Special care	Invasive ventilation	CPAP	High-flow oxygen	Oxygen support
Days of Intervention Median (Q1-Q3)	12 (5–37)	18 (8–31)	12 (0–26)	3 (0–26)	4 (1–10)	18 (0–28)	0 (0–3.5)

	**Outcomes for liveborn infants (n=22)**									
	Respiratory				Gastro	Infection	Neurological			
	RDS	BPD	Pneumothorax	PPHN	NEC	Sepsis	IVH Grade (n)			
Number of affected infants	19	6	2	3	3	18 suspected 2 confirmed	1 2	2 4	3 1	4 2

**Fig. 6:**
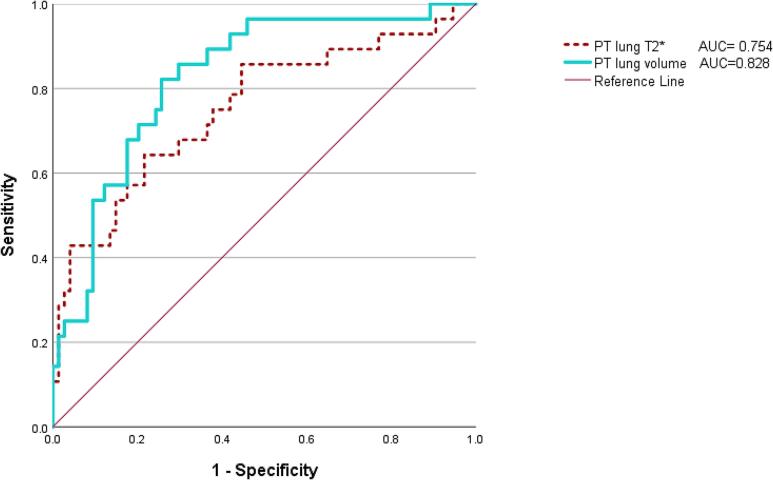
ROC curve of prediction of prematurity delivery based on lung volume and T2* values recorded from antenatal MRI scans.

Receiver Operating Curves for pulmonary mean T2* and pulmonary volume as a predictor of very PTB can be seen in Fig. 6 with area under the curves (AUC) being 0.754 and 0.828 respectively.

## Discussion

We have demonstrated that mean pulmonary T2* values are significantly lower in fetuses that subsequently delivered very preterm in comparison with fetuses that subsequently delivered at term. This finding was consistent for both lungs combined, the left and right lung and suggests functional pulmonary differences in those preterm fetuses. We have also confirmed that lung volumes were smaller in the preterm cohort which was again consistent for both lungs combined and the left and right lung. Low values of total lung volume and mean pulmonary T2* values were good predictors of subsequent very preterm delivery.

Although we have previously demonstrated that pulmonary volumes are lower in fetuses that subsequently delivered preterm [18], to our knowledge this is the first study to assess fetal lungs in a group of pregnancies that subsequently delivery very preterm using functional MRI techniques. T2* relaxometry is an advanced MRI imaging modality which utilises the fact that oxygenated and deoxygenated haemoglobin have different paramagnetic properties. T2* values are obtained from the combined effects of spin–spin relaxation (T2) and magnetic field inhomogeneities [31], but are sensitive to elements of tissue composition and structure providing an indirect assessment of tissue oxygenation or metabolic activity [31]. Limited fetal studies have previously utilised T2* relaxometry, however values assessed in the fetal brain [32] and placenta [29] have been reported to decrease in healthy pregnancies as gestation advances. We have previously published normative values of pulmonary mean T2* in uncomplicated pregnancies [27] between 20 and 38 weeks demonstrating that values increased with advancing GA, which may reflect increasing perfusion and metabolic requirements of pulmonary tissue as pregnancy progresses. In the current study we found that mean T2* values were constant in the control cohort, however this may reflect the narrower gestational age window assessed (20–32 weeks).

Lower pulmonary mean T2* values in the preterm cohort may be attributable to alterations in tissue composition, and vascularity and perfusion, all of which are known to affect mean T2* values [31]. Changes in metabolic activity associated with these developmental processes (ie altered angiogenesis and alveolar formation) in the antenatal preterm lung may therefore be reflected in the low values of both lung volume and mean T2* values. Infection and inflammation has been implicated [16], [33] in both the aetiology of sPTB [34], [35], [36] and pulmonary injury [37], [38], particularly at early gestations. Animal models have supported the role of infection as a causal pathway of preterm birth [39], [40]. Exogeneous administration of models of infection such as lipopolysaccharide, used in mouse models injected in the uterus or systemically, were found to promote preterm delivery [41], [42]. Direct intraamniotic infusion of IL-1β and TNF-α also triggers preterm labour in non-human primate models, highlighting the role of inflammation [43]. Human studies have also indicated that infection and inflammation may play a pivotal role in preterm delivery. Women with confirmed intra-amniotic infection or intrauterine inflammation diagnosed by raised interleukin levels and matrix degrading enzymes at amniocentesis at 18.9 weeks (median) gestation are at risk of preterm delivery [44]. Elevated cord levels of inflammatory cytokines interleukins IL-1β, IL-6, IL-8 and TNF-α have been reported in prematurely born infants [45], [46]. Animal models have also indicated that infection and inflammation may alter lung development in utero. Intrauterine infection spread to the lung in a sheep model culminating in injury and remodelling [47]. These changes have been found to be persistent affecting both vasculature [48] and lung morphology with a reduction in alveoli number and smooth muscle hypertrophy [48], [49], [50], [51]. Intra-uterine exposure of preterm sheep lungs to endotoxin with subsequent chorioamnionitis revealed alveolar and vascular simplification, thinner pleura and interlobular septa and a significant reduction in nonparenchymal volume (perivascular interstitium) when compared with control animals [52], [53]. In addition, inflammation has been reported to reduce levels of proangiogenic vascular endothelial growth factor (VEGF), a critical mediator of vascular formation [54]. VEGF levels have been shown to be reduced in umbilical cord blood and placenta samples in preterm born human infants where chorioamnionitis and funisitis were confirmed on placental histology [55]. Low levels of VEGF are associated with hindered alveolar development and subsequent alveolar simplification with loss of pulmonary capillaries [56].

The numbers included in this study were too small to assess differences between preterm fetuses with and without subsequent chorioamnionitis. Whilst 91 % of the preterm cohort had chorioamnionitis confirmed where placental histology was available (n = 23) with only two without confirmed infection; a comparison between those pregnancies with and without infection is not therefore possible. It is however possible that infection, inflammation and associated changes to lung morphology and vasculature may be responsible for the observed reduction in both lung volume and mean pulmonary T2* values. One of the major risk associated with PPROM is ascending infection of the amniotic cavity, particularly chorioamnionitis as 30–80 % of PPROM deliveries are complicated by chorioamnionitis [57]. Given the prolonged time between rupture of the membrane and delivery, it is therefore possible that chorioamnionitis might have already been present at the time of scan in the preterm cohort in the present study.

The reduction in mean pulmonary T2* values observed in the preterm cohort could also relate to alterations in the amount of fluid within the fetal lungs. Eighty-nine percent of fetuses in the preterm cohort of our study had oligohydramnios at the time of imaging. Oligohydramnios reduces the intrathoracic cavity size which increases the risk of respiratory complications and will inevitably alter the composition of tissue within of a region of interest [58], [59]. Between 8 and 26 % of fetuses will develop secondary pulmonary hypoplasia after oligohydramnios [60]. Alterations in fluid volume within the lung may also cause changes in pressure. Adequate pressure stretches the developing lung tissue and is necessary for normal lung growth and maturation [61]. Normal stretching has been shown to reduce collagen and elastin expression in animal model studies [62].

MRI studies in infants born preterm have demonstrated that prolonged fetal exposure to PPROM results in a reduction in total pulmonary volume with arrested alveolar development and a reduction in surface area [63], [64]. The numbers we investigated were too small to assess whether there was a difference between fetuses with and without ruptured membranes at the time of imaging, but this merits further investigation.

Lung volumes have previously been found to be good predictors of PTB [18]. This study not only confirms these findings (ROC AUC = 0.828, 95 % CI [0.741,0.915]) but also highlights the use of lung T2* as another factor to predict early delivery (ROC AUC = 0,754; 95 % CI [0.641,0.868]). Both AUC are statistically significant (p < 0.001).

### Strengths and limitations

To our knowledge this is the first study to utilise functional MRI techniques (e.g T2*) to evaluate lung development in a cohort of fetuses that subsequently delivered very preterm (<32 weeks). The application of DSVR post-processing pipelines facilitated pulmonary segmentation with correction for motion and distortion deterioration. We have previously demonstrated high inter and intra observer variability for segmentation of the lungs and T2* values used indicating this is a reliable and reproducible technique [25].

It should be noted that the sample size of this study is relatively small and there were significant differences between ethnic groups, GA at scan and BMI of the control and preterm groups. However, mitigation for this was incorporated into the regression modelling. Given there are multiple confounding factors such as suspected diagnosis of sepsis, variability in the days and type of ventilation in the neonatal period which may impact on pulmonary morbidity it was not possible to assess the correlation between mean T2* values and neonatal outcomes. A larger sample size would be required to explore this in the future.

### Conclusion

Our results indicate that alterations in pulmonary development associated with preterm birth may commence in the antenatal period. This study describes an MRI based approach on how to better explore those lung development changes and can have an impact on future respiratory complication and risk of preterm delivery. Additional work is needed to assess whether mean pulmonary T2* values can be used as a predictor of subsequent pulmonary morbidity associated with prematurity which may enhance counselling and care planning in the future.

## Declaration of competing interest

The authors have no conflicts of interest to declare.
